# Social intuition as a form of implicit learning: Sequences of body movements are learned less explicitly than letter sequences

**DOI:** 10.2478/v10053-008-0109-x

**Published:** 2012-05-21

**Authors:** Elisabeth Norman, Mark C. Price

**Affiliations:** 1 Faculty of Psychology, University of Bergen, Norway; 2 Haukeland University Hospital, Norway

**Keywords:** implicit learning, social intuition, intuition, artificial grammar learning, human movement, consciousness, fringe consciousness

## Abstract

In the current paper, we first evaluate the suitability of traditional serial reaction time (SRT) and artificial grammar learning (AGL) experiments for measuring implicit learning of social signals. We then report the results of a novel sequence learning task which combines aspects of the SRT and AGL paradigms to meet our suggested criteria for how implicit learning experiments can be adapted to increase their relevance to situations of social intuition. The sequences followed standard finite-state grammars. Sequence learning and consciousness of acquired knowledge were compared between 2 groups of 24 participants viewing either sequences of individually presented letters or sequences of body-posture pictures, which were described as series of yoga movements. Participants in both conditions showed above-chance classification accuracy, indicating that sequence learning had occurred in both stimulus conditions. This shows that sequence learning can still be found when learning procedures reflect the characteristics of social intuition. Rule awareness was measured using trial-by-trial evaluation of decision strategy (Dienes & Scott, 2005; Scott & Dienes, 2008). For letters, sequence classification was best on trials where participants reported responding on the basis of explicit rules or memory, indicating some explicit learning in this condition. For body-posture, classification was not above chance on these types of trial, but instead showed a trend to be best on those trials where participants reported that their responses were based on intuition, familiarity, or random choice, suggesting that learning was more implicit. Results therefore indicate that the use of traditional stimuli in research on sequence learning might underestimate the extent to which learning is implicit in domains such as social learning, contributing to ongoing debate about levels of conscious awareness in implicit learning.

## Introduction

Implicit learning is assumed to play a central role in various everyday behaviours. One example is the learning of complex patterns of motor responses involved in skills like playing musical instruments and driving, in which the details of the acquired knowledge are not fully accessible to conscious awareness (Clegg, DiGirolamo, & Keele, 1998). Another example is the acquisition of grammatical rules of one’s native language, which is claimed to occur largely independently of the conscious intent of the learner (Cleeremans, Destrebecqz, & Boyer, 1998; Reber, 1967, 1989). Yet another category of everyday behaviours explained in terms of implicit learning is the encoding and decoding of social signals in social interactions (Lieberman, 2000). For example, when people are sometimes able to accurately judge the personality of another person without being able to verbalize what the judgement was based on, this may be explained in terms of complex behavioural regularities being learned without full conscious awareness (Lewicki,Hill, & Czyzewska, 1992). Because of its assumed central role in social cognition, implicit learning has even been referred to as the cognitive substrate of social intuition (Lieberman, 2000). According to Lieberman, social intuition involves making rapid judgements about the emotions, personality, intentions, attitudes, and skills of others (p. 111). Such judgements are often based on the perception of sequences of various forms of nonverbal cues, including subtle facial expressions, body postures, and nonverbal gestures. Lieberman refers to this process as the “learning of nonverbal decoding.”This is regarded as an acquired ability that develops continuously throughout the life span.

The two most widely used implicit learning paradigms are primarily related to studying the first two of the above-mentioned areas of implicit learning: motor skill learning and language learning. In the serial reaction time (SRT) task, participants are first trained to make fast motor responses to indicate the shifting location of a target that moves between a fixed number of positions on a computer screen according to a complex repeating structure (Nissen & Bullemer, 1987). Learning is then measured in terms of reaction time (RT) increase when the sequence is violated. In the artificial grammar learning (AGL) task, participants are first presented with a series of letter strings in which the order of letters is, unbeknown to participants, governed by a complex finite-state grammar (Reber, 1967). In a subsequent test phase, participants are informed of the existence of a grammar, and asked to classify whether each of a series of novel letter strings follows this grammar or violates it.

These SRT and AGL paradigms have been modified to further increase their relevance and generalizability to everyday learning situations. For example, Witt and Willingham (2006) studied sequence learning for action sequences where responding to different target positions required different types of action (i.e., pressing, turning, pinching, or switching). The aim of this study was to address whether everyday action learning of, for instance, sequences of dance or martial arts movements, could be learned implicitly. The results supported the claim that learning of such sequences could indeed be associated with less than fully explicit learning. Similarly, Verwey’s (1994) discrete sequence production task has similarities to everyday implicit learning by virtue of involving extended practice over multiple test sessions. As an example of how AGL tasks may be applied to everyday learning contexts, Pacton, Perruchet, Fayol, and Cleeremans (2001) studied principles for natural language learning in children using methodological principles from AGL research in order to test whether children acquired knowledge of abstract rules.

In our view, it is less obvious how findings from standard SRT and AGL tasks can be applied to study the learning of nonverbal decoding of social signals. However, if this form of social intuition is one of the main real-life phenomena thought to be guided by implicit learning, it is important to consider the extent to which standard implicit learning experiments are appropriate for studying this phenomenon. We therefore suggest some methodological criteria for increasing the relevance of implicit learning experiments to situations of social intuition, and consider the extent to which the standard procedures used in SRT and AGL tasks meet these criteria. We then report an experiment which aimed to meet these methodological criteria. The real-world situation whose characteristics our experimental procedure attempts to reflect is the intuitive classification of different patterns of body movement.

### Increasing the relevance of implicit learning experiments to situations of social intuition

We suggest five methodological criteria for increasing the relevance of implicit learning experiments to situations of social intuition. After each suggestion, we briefly summarise the extent to which the requirement is already met by standard SRT and AGL procedures.

#### 1. Learning should involve exposure to stimulus sequences that represent a dynamic event.

Temporal sequences of events are considered to be a critical factor in perception of human body language. Drawing inferences about another person based on their body language is largely dependent on patterns of motion: “Bodies … are typically moving, and much of the information that bodies convey is in dynamic movement” (Slaughter, Stone, & Reed, 2004, p. 220). This is why it is easier to perceive gender, emotion, and direction of attention in a person that is moving compared to a person in static position (Slaughter et al., 2004). Learning to infer these states from body movement therefore involves sequence learning in the sense of repeated exposure to temporal sequences of different body postures.

##### The SRT task

In the SRT task, stimuli are indeed always presented in sequential order.

##### The AGL task

This is not necessarily the case in AGL experiments where stimuli are presented simultaneously. When stimuli are letters arranged in a string they may of course still be scanned sequentially since this is the way we read. However, sequential processing is less likely for picture stimuli that are not specifically arranged to encourage sequential scanning. Little is known about the relative ability to learn artificial grammars when nonverbal stimuli are employed which do not encourage sequential scanning to the same extent as letters.

#### 2. The sequence should involve different states of one entity rather than a series of different entities.

Nonverbal decoding of body movements, or for that matter facial expressions, involves observing changes in the state of a single entity which undergoes transformations in a sequential manner. The entity is the human body and the transformations are the movements and relative movements of the body parts (e.g., arms, legs, torso, etc.).

##### The SRT task

In the traditional version of the SRT task, the sequence indeed involves different states of the same entity, typically a geometrical shape (e.g., circle) that moves between different screen positions on different trials. However there are versions of the SRT task in which the sequence consists of different entities: In the category SRT task (Goschke & Bolte, 2007), participants respond to the identity of a series of different objects that are presented in random order, but the semantic categories of those objects follow a repeating sequence. A modified version of the SRT task where the colour and shape of the target vary randomly from trial to trial (Norman, Price, Duff, & Mentzoni, 2007) could be seen as yet another example of a sequence consisting of different entities.

##### The AGL task

In the traditional AGL task, each string of simultaneously-presented letters is an independent event (a new combination of letters) rather than a transformation of the string presented on the previous trial.

#### 3. Learning episodes should consist of many separate exemplars of the sequential regularity.

Learning of socially relevant behaviour patterns most often takes place over many separate episodes.

##### The SRT task

Here the same sequence is usually repeated continuously within one long learning block, rather than as separated presentations of sequences.

##### The AGL task

Here the letter strings, each of which is an exemplar of a “legal” sequence, are temporally distinct from each other.

#### 4. Repetition of exactly the same sequences should be minimized.

In everyday social interactions, examples of behaviour sequences that express the same emotion, motivation, or attitude will rarely be identical in every respect. In fact *social intuition*, defined in terms of implicit learning, is perhaps likely to be based on sequences that contain some inter-sequence variation since learning is likely to become more explicit when discrete exemplars of a behavioural regularity are always identical.

##### The SRT task

Standard “deterministic” SRT sequences do not contain deviations from the repeating sequence. However, in so-called probabilistic sequences the target violates the fixed sequence on a certain proportion of trials (Schvaneveldt & Gomez, 1998).

##### The AGL task

The sequences are not exact repetitions since they are determined by a complex finite-state grammar.

#### 5. The task should include precise measurement of what information participants are consciously aware of so that it is possible to discriminate between nonconscious implicit learning, social intuition, and explicit rule awareness.

Elsewhere (Norman, Price, & Duff, 2010; Price & Norman, 2008, 2009) we have argued that in order to identify learning that is neither fully unconscious nor fully conscious, but instead falls into the intermediate zone of “intuition”, “cognitive feelings,” or “fringe consciousness”, objective performance measures need to be supplemented by various subjective awareness measures. Therefore, any implicit learning experiment that aims to identify social intuition should include such measures.

##### The SRT and AGL tasks

One procedure for distinguishing between different levels of conscious rule awareness in implicit learning is to ask for confidence ratings after each classification response. According to the zero-correlation criterion, learning can be regarded as implicit when confidence is unrelated to classification accuracy (Dienes, Altmann, Kwan, & Goode, 1995). This procedure has been ap-plied both to SRT experiments (e.g., Norman et al., 2007) and to AGL experiments (e.g., Dienes et al., 1995). An additional procedure that has been developed within AGL experiments (Dienes & Scott, 2005; Scott & Dienes, 2008) but that has also been applied to SRT experiments (e.g., Fu, Dienes, & Fu, 2009) is to ask participants to report which *decision strategy* they used to arrive at their classification of a test sequence. If people are able to accurately classify letter strings on trials where they report that their classification was based on explicit memory or awareness of a rule (i.e., *explicit* decision strategies), it is assumed that the participants had conscious structural knowledge of the rules of the grammar on these trials (Dienes & Scott, 2005). If people are able to accurately classify letter strings on trials where they report that their decision was based on intuition, familiarity, or random choice (i.e., *implicit* decision strategies), it is assumed that structural knowledge was unconscious. This is the case even though participants may, on trials attributed to intuition or familiarity, have had conscious judgement knowledge of which sequences were grammatical. In other words, participants may have a conscious intuitive feeling for which sequences follow a rule even though they cannot consciously access the basis of this feeling (Norman et al., 2007, 2010; Price & Norman, 2008, 2009).

## Aims and design

In response to the methodological criteria outlined above, we conducted an experiment which combines aspects of both the SRT and the AGL paradigms in a novel manner. The experiment measured implicit learning of sequences of pictures of body postures that were presented individually but followed an artificial grammar structure. As in traditional SRT tasks, the sequences could be taken to represent a dynamic transformation of the state of a single entity (Suggestions 1 and 2). But as in traditional AGL tasks, participants were exposed to a series of discrete exemplars of the sequence regularity, and sequences were not completely identical to each other (Suggestions 3 and 4). The degree of learning was operationalised in terms of classification accuracy during a subsequent test phase where a series of novel sequences were presented. To distinguish between nonconscious, intuitive and fully explicit learning, the study also included detailed measurement of the degree of conscious awareness of acquired knowledge as commonly applied within SRT and AGL tasks (Suggestion 5).

We used pictures of body movements as stimuli, rather than, for example, the traditional letter strings employed in the AGL task, in order to improve the ecological validity of the study for understanding the role of implicit learning in social intuition. Our study therefore had similarities to the procedure recently used by Opacic, Stevens, and Tillmann (2009) who showed learning of sequences of body movement that followed an artificial grammar. Unlike Opacic et al. who presented participants with film sequences of modern dance, our stimuli were rapid sequences of still pictures. Static pictures are of course impoverished stimuli compared to the continuous body movements of real life, but sequences of static pictures of body movements are known to still support the perception of human action (Blake & Shiffrar, 2007) and are therefore relevant to studying the detection of patterns of body movement.

Using sequences of static pictures which can follow the kinds of rules used in traditional AGL studies also allowed us, unlike in the study by Opacic et al., to directly compare sequence learning of pictorial body posture stimuli with sequence learning in a comparison condition where pictures were substituted by traditional letter stimuli. This allowed us to address the extent to which the learning of standard finite-state grammars used in AGL generalises from highly familiar verbal language stimuli to the domain of nonverbal social intuition. Whether participants were trained and tested on sequences of pictures of body postures or sequences of letters was a between-subjects variable.

Opacic et al. (2009) did not include detailed measures of what information participants were consciously aware of in their study. In addition to introducing these kinds of measures into a study of sequence learning with nonverbal social stimuli, our own study is therefore novel in allowing a measurement of whether this degree of awareness is modified by stimulus type. If sequence learning with pictorial body movement stimuli turned out to be considerably more explicit than learning with letter stimuli, this would caution the extent to which even our modified sequence learning procedures provide support for the kind of nonverbal socially-relevant implicit learning suggested by Lieberman (2000). On the other hand, the body movement stimuli might be associated with a reduced tendency or ability to explicitly detect the sequence rule, suggesting that implicit learning is more robust in the nonverbal social domain than in sometimes controversial studies using letter strings or abstract sequences of dot positions.

## Method

### Participants, stimuli, and apparatus

Fourty-eight students (mixed sex) aged 20-39 years (*M* = 23.1) took part for a small financial renumeration.

Letter strings containing five to nine letters were taken directly from a published artificial grammar study by Scott and Dienes (2008, Experiment 1). Grammatical letter strings followed one of two finite-state grammars (Grammar A or Grammar B). There were 15 grammatical training strings for each grammar, and 30 grammatical test strings for each grammar (see Appendix A). The letters used were *T*, *V*, *X*, *R*, and *M*, written in black on a white background in Arial font. The letters were 2.8-3.4 cm wide and 3.7 cm high.

Body movement stimuli were five photographs of a person in different yoga positions (see Appendix B). These were arranged into sequences following exactly the same pattern as for letter stimuli. In other words, each of the five letters was simply replaced by one of the five photographs. Photos were purchased from www.fotolia.com and their background colour was modified to full white with Adobe Photoshop. On screen these stimuli subtended 4.0-7.5 cm in width and 8.8-13.5 cm in height.

The experiment was programmed in E-prime 2.0 (Schneider, Eschman, & Zuccolotto, 2002a, 2002b) on a Pentium 4 PC and displayed on a 19’’ Dell monitor at approximately 55 cm viewing distance. The experimenter gave a short introduction at the start of the experiment. All subsequent instructions and rating materials were presented on screen.

### Procedure

#### Training phase

Half the participants were assigned to view body posture stimuli and were informed that they would be presented with a number of yoga sequences that represented a new type of yoga called *mosho*. They were told to try and form an overall impression of the characterises of mosho-yoga. This will be referred to as the yoga condition. The other half of participants, in the letter condition, were told that they would be presented with a number of sequences of letters presented one at a time on the computer screen. To minimise conscious hypothesis-testing, all participants were told that the sequences would follow a pattern so complex that it was impossible to figure out consciously, and that they therefore should try to simply look at the sequences rather than search for a pattern.

The training phase consisted of three short blocks of 15 trials each. On each trial participants viewed a sequence of five to nine stimuli. Within the yoga and letter conditions, 12 participants viewed Gram-mar A sequences and 12 were shown Grammar B sequences. On each block each of the 15 training sequences were presented in random order. There was a short self-paced break between each block.

On each trial of the training phase, a central fixation cross was first presented for 1,000 ms, followed by a blank of 500 ms. Each sequence consisted of nine successive presentations of one screen display in each of nine positions on the screen. On the first display, a stimulus (letter or body photo) was presented to the far left for 700 ms then removed. On the second display, an object was presented in the second position from the left for 700 ms then removed, and so on. For sequences that were shorter than nine stimuli, the last screen displays contained blanks of 700 ms duration.

#### Test phase

At the start of the test phase, participants were informed that they would now be presented with a number of new sequences that they had not seen before.

Participants in the yoga condition were told that half of the yoga sequences would follow the mosho style and for each presented sequence they would have to decide whether they thought it was mosho or not. They pressed a key marked “Yes” ([8] on the numeric keypad) if they thought the sequence followed the yoga pattern, and they pressed a key marked “No” ([2] on the numeric keypad) if they thought the sequence did not. Participants in the letter condition were told that half of the sequences would follow the same pattern as the training sequences and their task was to identify those sequences, responding as for the yoga condition.

All participants viewed 60 sequences presented in one block. Half of the sequences were novel Grammar A sequences, and half were novel Grammar B sequences. Thus, for each participant half of the sequences were grammatical and the remainder were nongrammatical according to the grammar on which they had been trained. Presentation and timing of the sequences was as for the training phase. The order in which sequences were presented was randomised.

After each classification response, participants also had to indicate by a second key press whether they felt “Less confident” ([4] on the numeric keypad) or “More confident” ([6] on the numeric keypad). They were encouraged to distribute their responses evenly between the two categories to reduce response bias.

Participants then indicated the strategy they had used for their classification response. A subset of the categories developed by Scott and Dienes (2008) were used, namely *Random choice* (Key A), *Familiarity* (Key S), *Intuition* (Key D), *Rules* (Key F), and *Memory* (Key G). Response keys were clearly labelled with stickers. As part of the instructions the definitions of the various decision strategies were presented in writing on screen. The definitions were modified versions of those used by Scott and Dienes (2008) and were as follows: *Random choice* = the decision was completely random; *Familiarity* = the decision was based on some aspect of the sequence feeling familiar or unfamiliar; *Intuition* = the decision was based on a feeling or hunch that you to some extent trusted, but which you could not explain the basis of; *Rules* = the decision was based on whether you thought the sequence followed or violated one or more rules which you could state if asked; *Memory* = the decision was based on explicit memory for the whole sequence or parts of it. Shortened definitions of each decision strategy were presented on screen during every trial until a response had been made. The participant had to press a key to initiate the next trial.

## Results

Mean classification accuracy, as measured by the proportion of trials on which a correct response was made, was significantly above the chance level of .5 both in the yoga condition, *t*(21) = 2.50, *p* = .02 (two-tailed), and in the letter condition, *t*(23) = 4.76, *p* < .001 (two-tailed). A 2 × 2 ANOVA compared classification accuracy between these two conditions and between participants trained on Grammars A or B. Since training on Grammar A versus B had no significant main effect (*p* = .45)and did not interact with the yoga/letter manipulation (*p* = .36), this variable was excluded from subsequent analyses. However, mean classification accuracy was significantly higher in the letter condition (*M* = 0.59, *SE* = 0.02) than in the yoga condition, *M* = 0.53, *SE* = 0.01, *F*(1, 42) = 6.39, p = .02.

**Figure 1.**
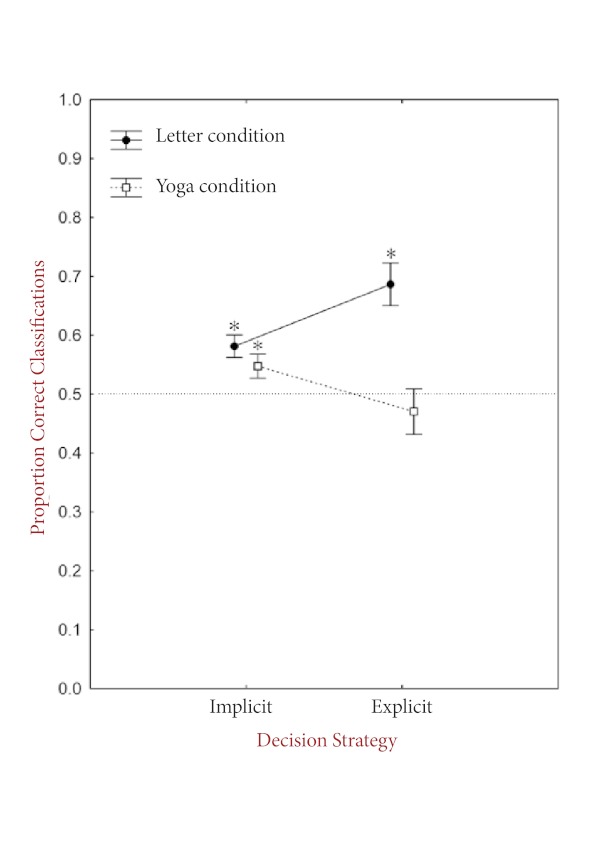
Mean classification accuracy (+/- *SE*) for responses attributed to implicit versus explicit decision strategies, plotted separately for the yoga and control conditions with chance level (.5) indicated. Asterisks indicate where performance is significantly above chance level.

Following Dienes and Scott (2005), trials were split into those attributed to implicit decision strategies (i.e., to intuition, familiarity, or random choice), and those attributed to explicit decision strategies (i.e., rules or memory). The absolute distribution of implicit versus explicit responses (approximately 70% versus 30%) did not differ across stimulus conditions, *F*(1, 44) = 1.52, *p* = .22. Overall, classification accuracy did not differ between trials attributed to implicit strategies (*M* = 0.56, *SE* = 0.01) and trials attributed to explicit strategies, *M* = 0.58, *SE* = 0.03,*F*(1, 41) = 0.26, *p* = .61. However, classification accuracy interacted significantly between stimulus condition and decision strategy, *F*(1, 41) = 10.75, *p* < .01 (see Figure 1). A post-hoc analysis (Fisher’s LSD test) showed that in the yoga condition there was a near-significant trend (*p* = .07) for classification accuracy to be higher for implicit (*M* = 0.55, *SE* = 0.02) than for explicit responses (*M* = 0.47, *SE* = 0.04). The opposite pattern was found in the letter condition, where accuracy was significantly higher (*p* < .01) for explicit (*M* = 0.69, *SE* = 0.04) than for implicit responses (*M* = 0.58, *SE* = 0.02). A comparison across the two stimulus conditions showed that for implicit responses, classification accuracy did not differ between the yoga and the letter condition (*p* = .43). However, for explicit responses, classification accuracy was significantly higher in the letter than in the yoga condition (*p* < .001). Comparison to chance (.5) showed that in the yoga condition, performance was significantly above chance level for implicit responses, *t*(21) = 2.59, *p* = .02 (two-tailed). However, it was not different from chance level for explicit responses, *t*(19) = -0.82, *p* = .42 (two-tailed), where the mean difference score was numerically below chance. In the letter condition, classification accuracy was significantly above chance level for both explicit, *t*(22) = 4.89, *p* < .001 (two-tailed), and implicit responses, *t*(23) = 4.34, *p* < .001 (two-tailed).

Classification accuracy was significantly higher on high-confidence (*M* = 0.60, *SE* = 0.02) than on low-confidence trials, *M* = 0.53, *SE* = 0.01, *F*(1, 44) = 12.57, *p* < .001, but was above chance for both high-, *t*(45) = 5.48, *p* < .001 (two-tailed), and low-confidence trials, *t*(45) = 2.38, *p* = .02 (two-tailed). This pattern was not influenced by stimulus condition.

Note that the above analyses exclude two outlier participants in the yoga condition whose classification accuracy was more than two standard deviations above their group mean. When these participants were included, the main effect of stimulus condition on overall classification accuracy failed to reach significance (*p* = .13). However, the significant interaction between stimulus condition and decision strategy remained significant, *F*(1, 43) = 7.45, *p* < .01. Even though the trend in the yoga condition for performance to be better for implicit than explicit responses was no longer present (*p* = .23), comparison of yoga performance to chance level showed the same pattern as before, with performance being significantly above chance for implicit, *M* = 0.55 , *SE* = 0.02, *t*(23) = 3.11, *p* < .01, but not for explicit responses, *M* = 0.51, *SE* = 0.04, *t*(21) = 0.15, *p* = .88. Classification accuracy remained significantly better on high-confidence (*M* = 0.61, *SE* = 0.02) than on low-confidence trials, *M* = 0.54, *SE* = 0.01, *F*(1, 46) = 14.98, *p* < .001, and was still above chance for both high-confidence, *t*(47) = 5.82,*p* < .001 (two-tailed), and low-confidence trials, *t*(47) = 2.79, *p* < .01 (two-tailed). This pattern was not influenced by stimulus condition.

## Discussion

The amount of learning and the degree of consciousness over what was learned was explored in each of two experimental conditions (yoga sequences vs. letter sequences) in an implicit learning experiment that combined procedural aspects of both the SRT and AGL tasks. During training, participants viewed sequences of stimuli that followed a rule based structure. Several aspects of the general procedure and stimulus design were intended to improve the suitability of the implicit learning experiment as an analog of learning involved in social intuition, namely: (a) the dynamic nature of the sequences, in which sequence elements appeared one by one; (b) the presentation of each sequence as a discrete exemplar; (c) the use of varying sequences that adhered to an artificial grammar rule rather than using cyclic repetition of an identical sequence. In addition, our yoga stimulus condition, which consisted of sequences of body postures, used more socially naturalistic stimuli than the letter sequences typically used in AGL tasks or the abstract shapes typically used in SRT tasks, and also depicted the transformation of a single entity (a body) rather than a juxtaposition of separate letters.

Participants in our yoga condition showed above chance learning of their artificial grammar. This complements the findings by Opacic et al. (2009) who showed learning of film sequences of body movement that followed an artificial grammar.

Unlike Opacic et al., we compared learning of body movement with learning of more traditional letter string sequences. Participants who learned letter sequences actually classified novel sequences more accurately than participants who learned yoga sequences. This differs somewhat from the findings of Pothos, Chater, and Ziori (2006), who found no difference in the learning of artificial grammars when stimuli were either letter strings, arrangements of geometric shapes, or sequences of cities that corresponded to the routes of a travelling salesman. However, none of the stimulus conditions in the study of Pothos et al. were as complex as pictures of body posture, and it is possible that the increased complexity of our more naturalistic stimuli reduced sequence learning. More learning in the letter condition could also be explained in terms of differences in prior familiarity with the two types of stimuli. In an AGL task using novel geometrical symbols as stimuli, Scott and Dienes (2010) already showed that prior familiarisation with the symbols increased classification accuracy. However, the relative familiarity of letters versus other types of stimuli does not seem to have influenced relative performance in the study by Pothos et al. (2006). It should also be kept in mind that two high scoring outliers were dropped from the yoga condition in our study and without their exclusion the group difference we found would not reach significance. The most conservative interpretation of our data is therefore that, like others, we have been able to show that more naturalistic stimuli can yield at least some learning in studies of this kind.

Our study included two measures of conscious awareness, namely confidence ratings and trial-by-trial evaluations of decision strategy. We found that the tendency of participants to attribute their classification responses to explicit decision strategies (rules or memory) on about 30% of trials, versus implicit decision strategies (intuition, familiarity, or random choice) on about 70% of trials, did not differ with stimulus type. However classification accuracy for trials attributed to implicit versus explicit strategies did show a significant interaction with stimulus type. A comparison within each experimental condition of relative classification accuracy for trials attributed to implicit versus explicit strategies can be used to draw inferences about the degree of conscious awareness of the learned sequence structure: If participants show higher classification accuracy for explicit than for implicit trials, this suggests that an explicit approach, where participants apply conscious rules and memories, is more beneficial than an intuitive approach where they respond on the basis of familiarity, intuition, or random choice. Such a pattern indicates that participants’ conscious rules and memories accurately reflect the sequence structure , that is, learning is associated with some conscious awareness of the sequence rule. However, if performance is better on implicit than on explicit trials, this suggests that participants have not developed accurate conscious structural knowledge of the rule set. In the current study we found that participants in the letter condition showed a significant tendency to perform better on trials attributed to explicit strategies than to implicit strategies, even though classification was above chance for both subsets of trials. Participants in the yoga condition showed a trend in the opposite direction with classification accuracy only reaching significantly above chance for implicitly attributed trials. For explicit trials, performance did not differ from chance, but the mean score was in fact slightly below chance level. This indicates that correct classification was mediated largely by implicit processing in the yoga condition but that at least some degree of explicit learning developed in the letter condition. Comparisons across stimulus conditions confirmed that participants in the letter condition showed an advantage over participants in the yoga condition for trials attributed to explicit, but not to implicit decision strategies. The overall picture that emerges from the current set of results indicates that sequences of body movements appear to be learned less explicitly than sequences of letters.

Could the tendency for successful conscious hypothesis testing in the letter condition, as opposed to a more passive but still successful use of intuitive feelings in the yoga condition, be explained in terms of the two types of stimuli being associated with different levels of familiarity? For example, more familiar stimuli may place less load on working memory, making it easier to intentionally examine the sequence structure. However, Scott and Dienes (2010) found that even though prior familiarisation with symbols increased overall classification accuracy in an AGL task, the influence of familiarity did not influence the relative proportion of responses attributed to implicit versus explicit strategies, and did not have a differential impact on classification accuracy during these two types of trials. Familiarity differences are therefore unlikely to explain the difference between our yoga and letter results.

Instead we suggest it is more likely that the interaction between stimulus condition and decision strategy is related to the visual versus verbal character and relative complexity of our two groups of stimuli. Sequences of separate letter stimuli can be categorised and rehearsed in working memory in terms of their well-learned verbal codes. However, sequences of body movements that now consist of apparent transformations of the same entity lack pre-existing verbal codes for each sequence item and will therefore be more difficult to explicitly memorise. This will leave less resources available for conscious hypothesis testing in the yoga than in a letter condition. The fact that participants in the yoga condition may distribute their attention across various aspects of their more complex stimuli, such as the face, hair, clothes and so on, will further reduce the ease with which sequence patterns can be detected. Any conscious hypothesis-testing that they do engage in might even relate to stimulus aspects other than body posture. Classification judgements that are attributed to explicit rules or memory of specific stimulus characteristics are therefore likely to be based on spurious clues and performance will be poor on those trials. This might explain why participants in the yoga condition did not perform above chance on trials attributed to explicit strategies. When participants refrained from trying to use explicit knowledge, but just followed their intuitions as they might do in the real-life context of observing body movements, performance seemed better. This interpretation is consistent with findings of Norman et al. (2007) who found that increasing the complexity of stimuli in the SRT task by adding random variation in the colour and shape of target stimuli seems to give rise to a less explicit and more implicit style of learning than reported in some previous studies(e.g., Wilkinson & Shanks, 2004). Note that other studies have also investigated the effects of complexity on implicit learning by varying properties of the learned rule or by comparing performance under single versus dual task conditions (see Frensch, Buchner, & Lin, 1994; Frensch, Lin, & Buchner, 1998).

Since implicit decision strategies were defined to include not just intuition and familiarity but also random responses, it could be argued that the yoga participants’ advantage for implicit over explicit trials might derive from non-conscious orientation responses rather than from conscious feelings. In other words, their judgement knowledge (Dienes & Scott, 2005) may have been either non-conscious or conscious. One aspect of the data which may indicate that judgement knowledge was more conscious is that the relation of confidence ratings to classification performance was equivalent in our yoga and letter conditions. What participants learned in the yoga condition is therefore best understood as a cognitive feeling (Price & Norman, 2008, 2009) a variety of “fringe consciousness” where conscious feelings are experienced and can be used to guide behaviour and judgements, including confidence ratings, in the absence of full conscious awareness of the information-processing antecedents of those feelings (Norman et al., 2007, 2010; Price & Norman, 2008, 2009). Hence, the apparent discrepancy between our *decision strategy* and *confidence rating* awareness measures with the former but not the latter showing a modulation of explicit knowledge by stimulus condition could well be related to the distinction between structural and judgement knowledge.

In conclusion, we have argued that sequence learning experiments can be constructed in a manner that simulates the properties of real-world social learning environments better than traditional SRT and AGL paradigms, and we have shown that sequences based on artificial grammars can still be learned under these conditions. We have also found that the learning obtained under these conditions appears to be based more on explicit rule knowledge when sequence elements are letters, but based more on implicit intuitive feelings when elements are images of body posture. Perhaps the most important implication of our findings is that researchers of implicit learning may underestimate the possibility and real-world prevalence of truly implicit learning if they restrict themselves to using stimuli such as letter sequences or sequences of simple geometrical shapes. Given the ongoing controversy over whether complex rules are learned via explicit knowledge of rule fragments (e.g., Wilkinson & Shanks, 2004), via conscious intuitions (e.g., Norman et al., 2007; Scott & Dienes, 2008), or entirely non-consciously (e.g., Destrebecqz & Cleeremans, 2001), attention should also be given to the importance of stimulus materials. Further research is now needed to identify the relative importance of stimulus complexity per se versus the extent to which stimuli are naturalistic and socially relevant.

## Figures and Tables

**Figure B1.**
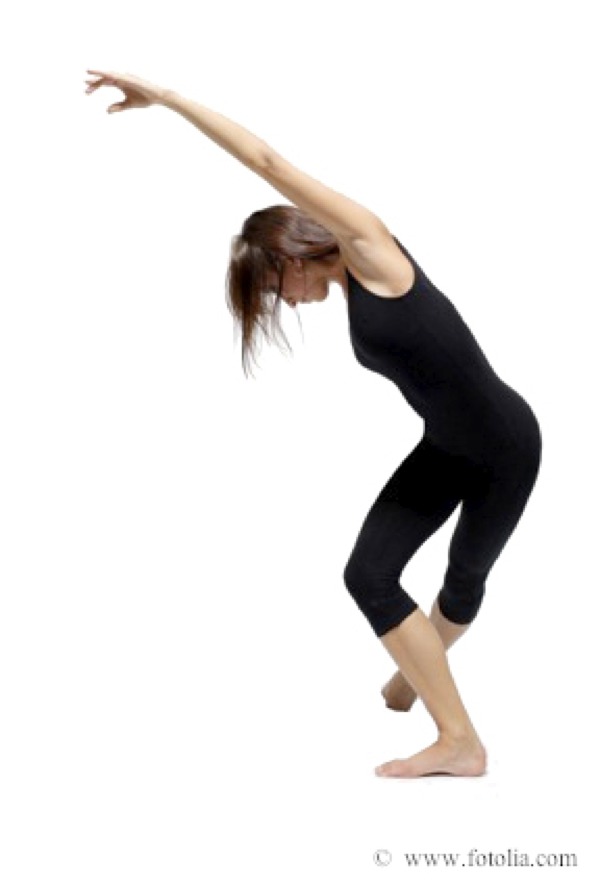
Letter *M*. Photo purchased from www.fotolia.com

**Figure B2.**
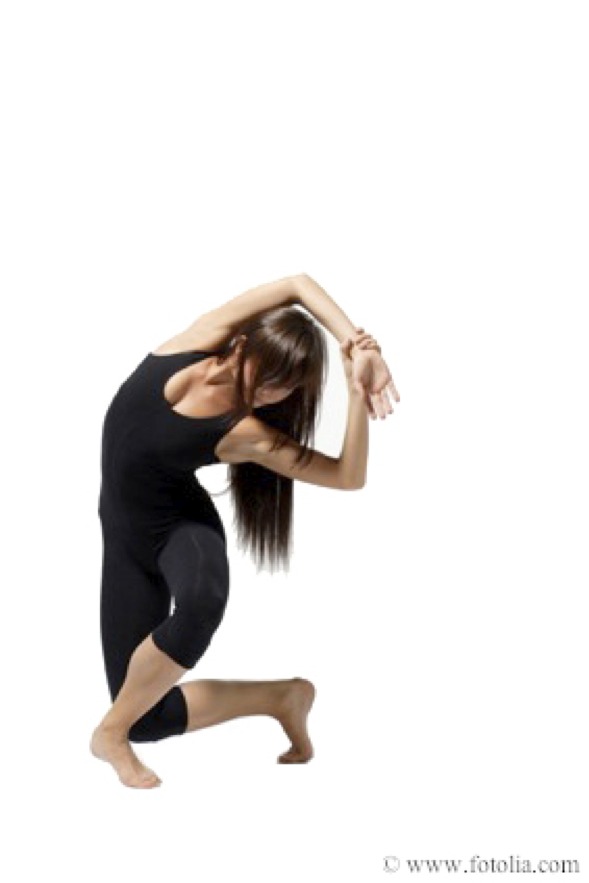
Letter *R*. Photo purchased from www.fotolia.com

**Figure B3.**
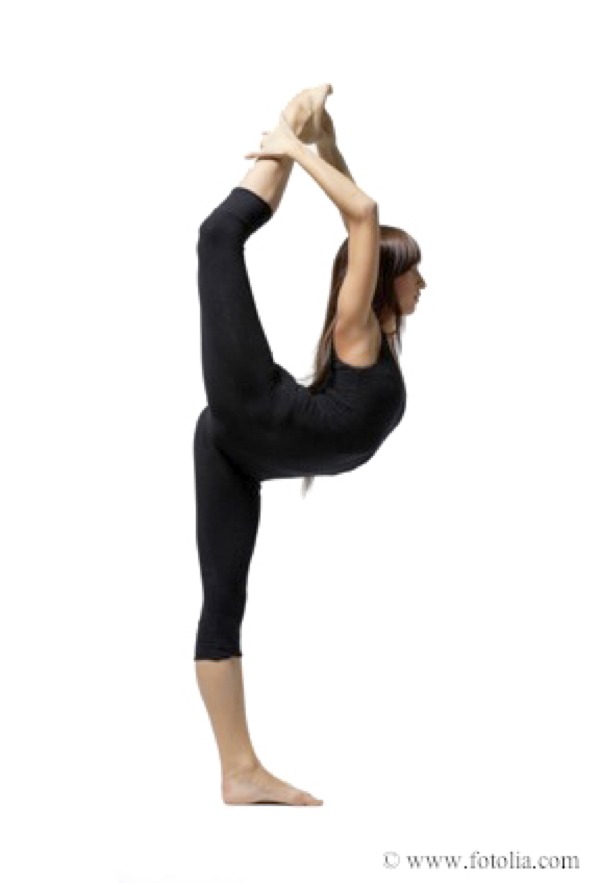
Letter *T*. Photo purchased from www.fotolia.com

**Figure B4.**
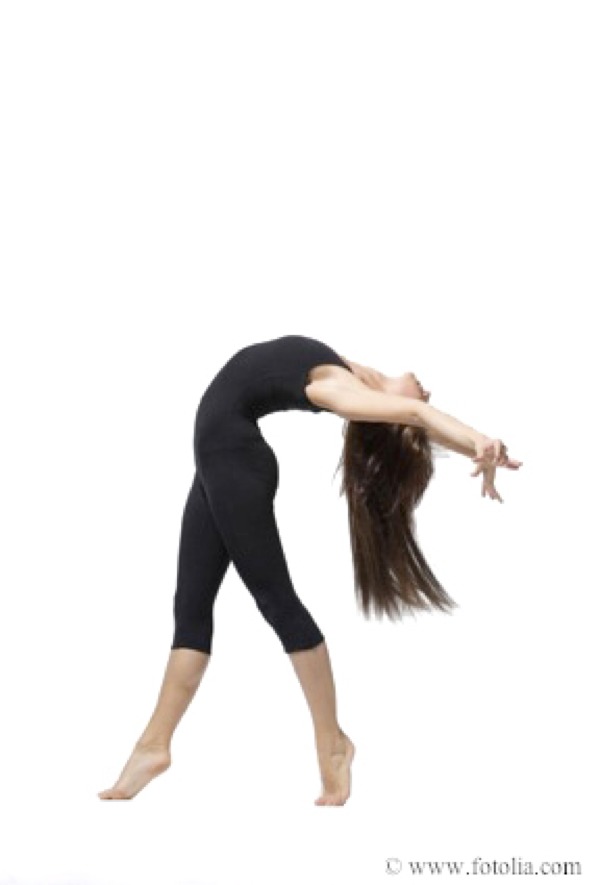
Letter *V*. Photo purchased from www.fotolia.com

**Figure B5.**
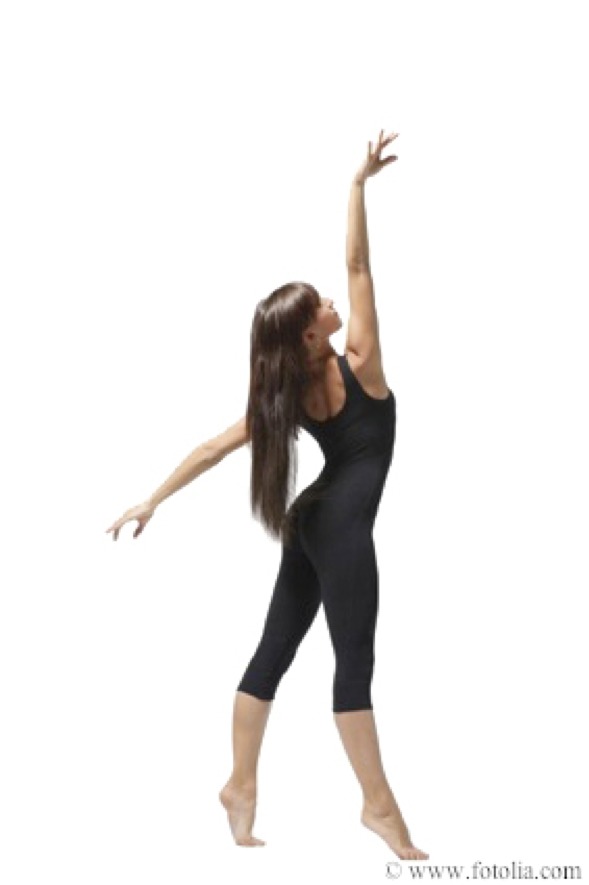
Letter *X*. Photo purchased from www.fotolia.com

## Appendix A

### Letter strings

**Table:** Training strings: Grammar A

XMMXM
XXRVTM
VVTRVM
VTTTTVM
XMMMMXM
XXRTVTM
XMXRTVTM
XXRTTTVM
XMMXRTVM
VTVTRVTM
VVTRTTVTM
VTTTVTRVM
XMXRTTTVM
XMMMXRVTM
XMMMXRTVM

**Table:** Training strings: Grammar B

XXRRM
VVRXRM
XXRRRM
VTRRRRM
VVTRXRM
XMVTRXM
VVRXRRRM
VVTRXRRM
XMVRMTRM
XMVTRMTM
VVTTTRMTM
VVTTTRXRM
XMVRMTRRM
XMVTRMTRM
XMVTTRMTM

**Table:** Test strings: Grammar A

VTVTM
XXRVM
VTTTVM
VTTVTM
XMMMXM
XMXRVM
VTTTVTM
VTVTRVM
VVTRVTM
XMMXRVM
XMXRTVM
XXRTTVM
VTTTTVTM
VTTVTRVM
VTVTRTVM
VVTRTVTM
XMMMXRVM
XMXRTTVM
XXRTTVTM
XXRVTRVM
VTTTVTRVM
VTTVTRTVM
VTVTRTTVM
VTVTRTVTM
VVTRTTVTM
XMMXRTTVM
XMXRTTVTM
XMXRVTRVM
XXRTTTVTM
XXRTVTRVM

**Table:** Test strings: Grammar B

VTRRM
XMTRM
VTRRRM
VVRMTM
XMTRRM
XMVRXM
VVRMTRM
VVRXRRM
VVTRMTM
XMTRRRM
XMVRMTM
XMVRXRM
VTRRRRRM
VVRMTRRM
VVRMVRXM
VVTTRMTM
VVTTRXRM
XMVRXRRM
XMVTRXRM
XMVTTRXM
VVRMVRXRM
VVRMVTRXM
VVTRMTRRM
VVTRMVRXM
VVTRXRRRM
VVTTRMTRM
VVTTRXRRM
XMVRMVRXM
XMVRXRRRM
XMVTRXRRM
